# Trajectories of physical functioning and its predictors in older adults: A 16-year longitudinal study in China

**DOI:** 10.3389/fpubh.2022.923767

**Published:** 2022-08-25

**Authors:** Yinan Zhao, Yunzhu Duan, Hui Feng, Jiahui Nan, Xiaoyang Li, Hongyu Zhang, Lily Dongxia Xiao

**Affiliations:** ^1^Xiangya School of Nursing, Central South University, Changsha, China; ^2^Xiangya-Oceanwide Health Management Research Institute, Central South University, Changsha, China; ^3^National Clinical Research Center for Geriatric Disorders, Xiangya Hospital, Changsha, China; ^4^College of Nursing and Health Sciences, Flinders University, Adelaide, SA, Australia

**Keywords:** physical function, older people, longitudinal survey, trajectory, predictors

## Abstract

**Objective:**

Maintaining and delaying a decline in physical function in older adults is critical for healthy aging. This study aimed to explore trajectories, critical points of the trajectory changes, and predictors among older people in the Chinese community.

**Design:**

This study was one with a longitudinal design performed in China.

**Setting and participants:**

The target population was community-dwelling older adults aged over 65 years. A total of 2,503 older adults from the Chinese Longitudinal Healthy Longevity Survey (CLHLS) were included in this study.

**Methods:**

Physical functioning was measured by instrumental activities of daily living (IADL). Population-based trajectory models were used to identify potential heterogeneity in longitudinal changes over 16 years and to investigate associations between baseline predictors and different trajectories for different cohort members using LASSO regression and logistic regression.

**Results:**

Four trajectories of physical function were identified: slow decline (33.0%), poor function and moderate decline (8.1%), rapid decline (23.5%), and stable function (35.4%). Older age, male sex, worse self-reported health status, worse vision status, more chronic diseases, worse cognitive function, and a decreased frequency of leisure activity influenced changes in the trajectory of physical function. Having fewer teeth, stronger depressive symptoms, a lack of exercise, and reduced hearing may increase the rate of decline.

**Conclusion and implications:**

Four trajectories of physical function were identified in the Chinese elderly population. Early prevention or intervention of the determinants of these trajectories can maintain or delay the rate of decline in physical function and improve healthy aging.

## Introduction

With the rapid aging of the population, significant challenges arise from the sheer diversity of health and functional states of older people (1). In China, the aging population, those aged 80 years or over, has led to a sharp increase in the number of disabled older people (2). As a result, China's annual financial demand for long-term care has surged and is expected to reach 8,530.8 billion yuan by 2050 (2).

Physical function is often measured by the basic activities of daily living (ADL) and the instrumental activities of daily living (IADL) (3, 4). ADL refers to activities for self-care, which are fundamental to living in society. In contrast, IADL refers to activities supporting daily life within the home and community and are more concerned with self-reliant functioning in a given environment and often require more complex interactions (5). Therefore, ADL disability is more suitable for identifying people with severe functional losses, most of whom are care-dependent. In contrast, IADL is more suitable for identifying people with functional decline, most of whom are at high risk of becoming care-dependent (1, 6).

The trajectories of change in physical function and associated influences are the first steps in the study of older adult health. In recent years, research has emerged on the potential determinants of the functional states of older people. The research results show that, from a clinical or public education perspective, it would make sense that sociodemographic, health, and lifestyle factors are associated with functional status and may cause people to follow specific trajectories of physical functional decline (7–12). It is worth noting that there are many modifiable lifestyle risk factors, such as smoking, alcohol abuse, sedentary behavior, poor sleep, and poor dietary habits (8, 9, 11–22). However, existing studies focusing on lifestyle and disability have several shortcomings, including (1) focusing only on special populations with functional decline in some domains; (2) focusing only on one behavior; (3) short follow-up periods; and (4) using ADL disability as the primary outcome but lacking evidence regarding IADL disability as the primary outcome.

The trajectory analysis of physical function in older people, which is important for determining whether a trajectory occurs and providing some support at key points of the trajectory, has potential benefits for maintaining intrinsic capacity and reducing the incidence of disability and care dependency. However, research on the most significant variables among the multifaceted influences by machine learning methods for different trajectories of physical functional decline is lacking. Accordingly, this study aimed to explore the trajectories and critical points of the trajectory changes and the relationship between sociodemographic characteristics, sociopsychological factors, and lifestyles among older people in the Chinese community.

## Methods

### Design and study sample

All data were obtained from the Chinese Longitudinal Healthy Longevity Survey (CLHLS) database. The CLHLS is an ongoing study of the physical, emotional, cognitive, social functioning, lifestyle, and environment of older people in China. It is one of the largest research studies on the health and related factors of older people in China and is based on a nationally representative sample (23). We excluded older people who had missing values for two or more waves. A total of 2,503 samples were included according to the selection criteria. This study followed the GRoLTS checklist (24).

### Measurement

#### Physical function

Physical function was measured by instrumental activities of daily living (IADL) and ADL. IADL was rated using eight questions, including the ability to visit neighbors, cook a meal, go shopping, wash clothes, walk 1 km at a time, lift a weight of 5 kg, continuously squat and stand three times, and take public transportation. ADL was measured across six subdomains, namely, bathing, dressing, toileting, indoor moving, continence of defecation, and eating. The CLHLS sample showed good internal consistency in ADL/IADL, with a Cronbach's α of 0.818 (25).

#### Health-related information

The general health information included perceptual function (vision, hearing), the number of natural teeth, and chronic diseases. The self-rated health status was assessed using the question “How do you feel about your health?” Self-rated quality-of-life was assessed using the question “How do you feel about your quality of life?” The emotional status was assessed with two questions: “Do you feel fearful or anxious?” and “Do you feel lonely and isolated?”. CMMSE was used to assess the cognitive function; in this study, the internal consistency of CMMSE was 0.808 (26, 27).

#### Lifestyle assessment

In Jin's study, five lifestyle factors, namely, smoking, alcohol consumption, exercise status, diet and mental health (28) were examined, and a lifestyle index was created. The researchers asked participants four questions about four foods (vegetables, fruits, meat, and eggs) and the frequency at which they were consumed. Participants were defined as consuming a healthy diet if they answered “almost every day” or “not every day, but at least once a week” for at least two of the four foods (vegetables, fruits, meat, and eggs).

#### Leisure activities

Leisure activities were assessed using a total of eight questions: “Do you do housework at present?”, “Do you do any outdoor activities at present?”, “Do you do garden work?”, “Do you read newspapers/books at present?”, “Do you raise domestic animals/pets at present?”, “Do you play cards/mah-jong at present?”, and “Do you watch TV or listen to the radio at present?” Each question had five answers: almost every day (score of 1), not daily but once per week (score of 2), not weekly (score of 3) but at least once per month (score of 4), and not monthly but sometimes or never (score of 5). The sum of the eight activities ranged from 8 to 40, with a low score representing a high frequency of leisure activities (29).

#### Covariates

All covariates were obtained at baseline and included age, gender (male or female), residence (city, town and rural), years of school, and current marital status (currently married, separated, divorced, widowed, never married).

### Statistical analysis

Participants' physical function trajectories were modeled using a group-based trajectory model (GBTM). A GBTM is a type of latent class growth model used to identify clusters of individuals who follow similar developmental trajectories on outcomes of interest by fitting a semiparametric mixture model to longitudinal data using a maximum likelihood estimate and is widely used in clinical research (30, 31).

The most predictive features among the fundamental characteristics in the primary dataset were selected by performing least absolute shrinkage and selection operator (LASSO) regression using R (https://www.r-project.org/), which is very suitable for the shrinkage of a high-dimensional dataset. The aim of this analysis is to explore the predictors between different trajectories. We considered a multivariable prediction model obtained by multinomial logistic regression to explore the predictors between different trajectories. A two-sided *p* < 0.05 was considered statistically significant. We analyzed the dataset using multivariate interpolation of chained equations (MICEs). MICE adds new functionality for imputing multilevel data, automatic predictor selection, data handling, post-processing imputed values, specialized pooling routines, model selection tools, and diagnostic graphs. Imputation of categorical data is improved in order to bypass problems caused by perfect prediction (32).

## Results

### Characteristics of the study samples

**Figure 1:**
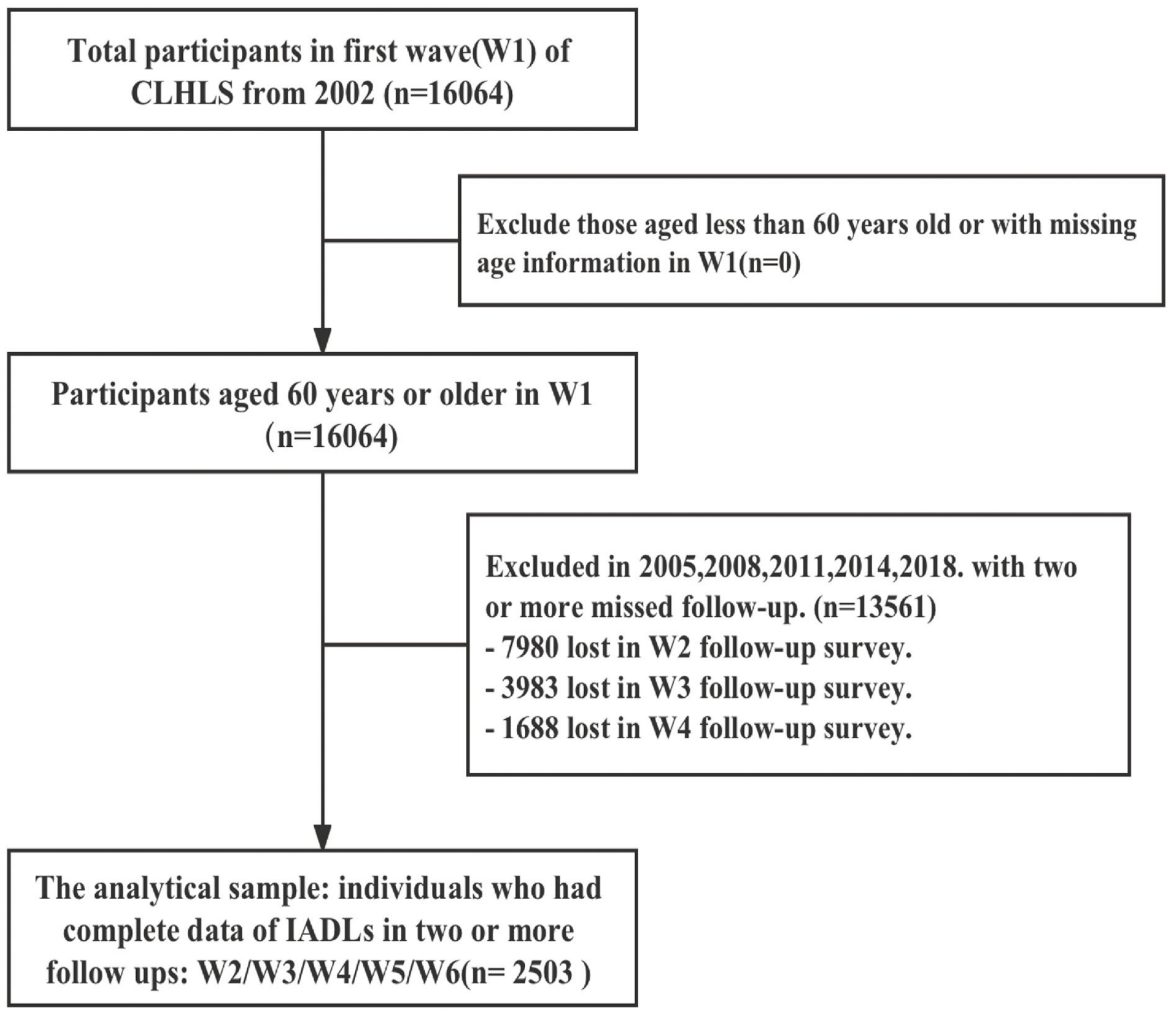
Flowchart of the study population.

To obtain the most accurate results, all six waves of subjects were included in this study, with the sample size adjusted to the number of subjects who were excluded. The participants in the final analysis (*n* = 2,503) were younger, more educated, had a better marital status, and were less lonely and isolated (all *p* < 0.05; Figure 1; Table 1). At baseline, the age of the study participants ranged from 65 to 104 years, with an average age of 74.71 years (SD 7.81). The number of males and females was balanced (1,177 vs. 1,326). Most of the older people lived in rural areas (61.8%). Nearly half of the older people did not complete primary education. The baseline characteristics of the participants for different trajectory groups are shown in Table 2.

**Table 1:** Baseline characteristics between participants and non-participants.

**Variable**	**Participants**	**Non-participants**
	**(*N* = 2,503)**	**(*N* = 13,561)**
Age, *M* (SD)	74.71 (7.81)	88.47 (11.02)
**Gender, (** ***n*** **, %)**		
Male	1,177 (47.0)	7,893 (58.2)
Female	1,326 (53.0)	5,668 (41.8)
**Residence, (** ***n*** **, %)**		
City	421 (16.8)	3,424 (25.3)
Town	534 (21.3)	3,015 (22.2)
Rural	1,548 (61.8)	7,122 (52.5)
Years of schooling, *M* (SD)	2.70 (3.58)	2.63 (8.74)
**Current marital status, (** ***n*** **, %)**		
Currently married	1,357 (54.2)	3,385 (25.0)
Separated	69 (2.8)	227 (1.7)
Divorced	19 (0.8)	75 (0.6)
Widowed	1,039 (41.5)	9,692 (71.5)
Never married	10 (0.8)	182 (1.3)
Number of natural teeth, *M* (SD)	14.07 (10.78)	7.48 (9.41)
Number of chronic diseases, *M* (SD)	0.80 (1.01)	0.92 (1.13)
ADL, *M* (SD)	6.13 (0.70)	7.40 (2.61)
MMSE score, *M* (SD)	18.75 (3.59)	15.51 (6.16)
Lifestyle, *M* (SD)	2.52 (1.00)	2.56 (0.93)
Smoke, *M* (SD)	0.75 (0.43)	0.83 (0.38)
Drink, *M* (SD)	0.76 (0.43)	0.80 (0.40)
Exercise, *M* (SD)	0.37 (0.48)	0.30 (0.46)
Diet, *M* (SD)	0.64 (0.48)	0.63 (0.48)
Depression symptoms, *M* (SD)	0.48 (0.72)	0.56 (0.77)
Self-reported quality of life, *M* (SD)	2.40 (0.98)	4.49 (1.56)
Self-reported health, *M* (SD)	2.47 (1.02)	4.39 (1.65)
Leisure activities, *M* (SD)	26.53 (5.35)	31.91 (6.44)

**Table 2:** Baseline characteristics of the total sample and the sample by the different trajectory groups.

**Variable**	**Total (*N* = 2,503)**	**Trajectory group**			
		**Stable** **(*N* = 905)**	**Slow decline** **(*N* = 817)**	**Poor function with moderate** **decline** **(*N* = 191)**	**Rapid decline** **(*N* = 590)**
Age, *M* (SD)	74.71 (7.81)	70.15 (4.87)	74.25 (6.44)	84.48 (8.43)	79.18 (7.71)
**Gender, (** ***n*** **, %)**					
Male	1,177 (47.0)	563 (62.2)	348 (42.6)	46 (24.1)	220 (37.3)
Female	1,326 (53.0)	342 (37.8)	469 (57.4)	145 (75.9)	370 (62.7)
**Residence, (** ***n*** **, %)**					
City	421 (16.8)	166 (18.3)	109 (13.3)	47 (24.6)	99 (16.8)
Town	534 (21.3)	184 (20.3)	174 (21.3)	42 (22.0)	134 (22.7)
Rural	1,548 (61.8)	555 (61.3)	590 (60.5)	102 (53.4)	357 (60.5)
Years of schooling, *M* (SD)	2.70 (3.58)	3.63 (5.05)	2.30 (5.63)	1.76 (7.59)	2.16 (6.56)
**Current marital status, (** ***n*** **, %)**					
Currently married	1,357 (54.2)	615 (68.0)	438 (53.6)	49 (25.7)	255 (43.2)
Separated	69 (2.8)	33 (3.6)	25 (3.1)	/	11 (1.9)
Divorced	19 (0.8)	8 (0.9)	10 (1.2)	/	1 (0.2)
Widowed	1,039 (41.5)	241 (26.6)	337 (41.2)	142 (74.3)	319 (54.1)
Never married	10 (0.8)	8 (0.9)	7 (0.9)	/	4 (0.7)
Number of natural teeth, *M* (SD)	14.07 (10.78)	17.97 (10.56)	14.14 (10.14)	6.58 (8.57)	10.40 (10.09)
Number of chronic diseases, *M* (SD)	0.80 (1.01)	0.63 (0.84)	0.82 (0.98)	1.26 (1.37)	0.89 (1.09)
ADL, *M* (SD)	6.13 (0.70)	6.02 (0.16)	6.07 (0.41)	6.93 (1.99)	6.12 (0.60)
IADL, *M* (SD)	22.59 (2.96)	23.86 (0.59)	22.86 (2.22)	16.38 (4.94)	22.23 (2.58)
MMSE score, *M* (SD)	18.75 (3.59)	19.90 (2.32)	18.59 (3.38)	16.45 (5.84)	17.95 (3.90)
Lifestyle, *M* (SD)	2.52 (1.00)	2.44 (1.05)	2.52 (1.00)	2.60 (0.89)	2.62 (0.95)
Smoke, *M* (SD)	0.75 (0.43)	0.67 (0.47)	0.77 (0.42)	0.86 (0.35)	0.81 (0.39)
Drink, *M* (SD)	0.76 (0.43)	0.68 (0.47)	0.78 (0.41)	0.87 (0.34)	0.81 (0.39)
Exercise, *M* (SD)	0.37 (0.48)	0.41 (0.49)	0.34 (0.47)	0.29 (0.45)	0.38 (0.49)
Diet, *M* (SD)	0.64 (0.48)	0.68 (0.47)	0.63 (0.48)	0.59 (0.49)	0.62 (0.49)
Depression symptoms, *M* (SD)	0.48 (0.72)	0.37 (0.65)	0.55 (0.77)	0.56 (0.75)	0.50 (0.72)
Self-reported quality of life, *M* (SD)	2.40 (0.98)	2.29 (0.79)	2.39 (0.88)	2.91 (1.78)	2.41 (0.97)
Self-reported health, *M* (SD)	2.47 (1.02)	2.22 (0.81)	2.51 (0.92)	3.19 (1.71)	2.55 (1.00)
Leisure activities, *M* (SD)	26.53 (5.35)	24.65 (4.86)	26.64 (4.88)	32.00 (5.65)	27.50 (5.10)

ADL, activities of daily living; IADL, instrumental activities of daily living; MMSE, the Mini-Mental State Exam.

### Physical function trajectory models

**Figure 2:**
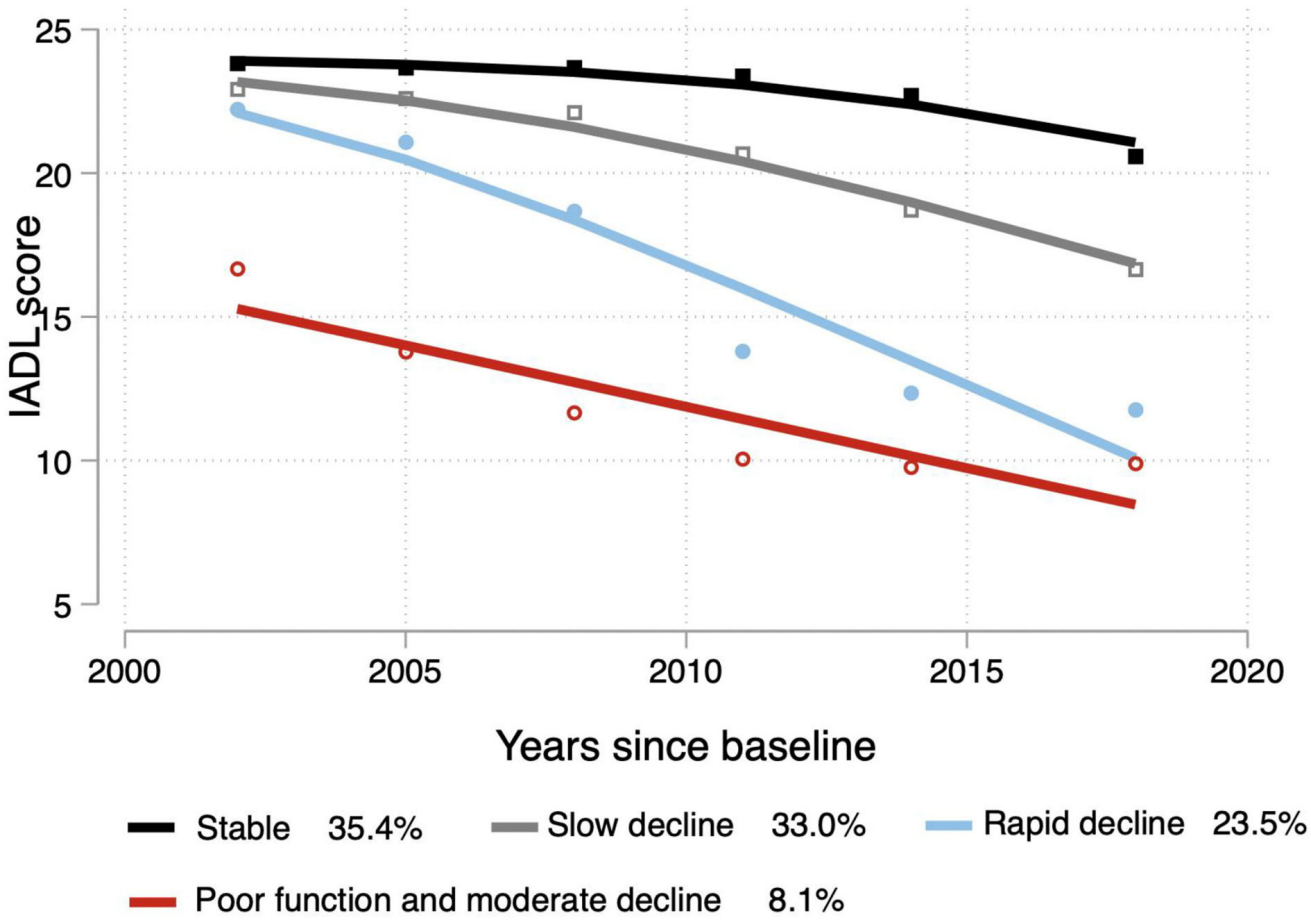
Trajectories of the IADL scores. The solid lines (black: stable; gray: slow decline; blue: rapid decline; red: poor function and moderate decline) represent estimated values.

After several data processing sessions, we found that among the models in groups 1–4, the absolute BIC values of the models in group 4 were lower than those of the other models, and the average posterior probability (Ave PP) values were high, all being greater than 0.8. A comparison of the models identified by GBTM for 1–4 trajectory classes is reported in Table 3. There were four different trajectory groups in which the model reached optimal values and maintained clinical applicability (Figure 2). The first group consisted of 35.4% of the population, marked as “stable.” The second cohort consisted of 33.0% of the population, labeled “slow decline.” The third cohort consisted of 23.5% of the population, labeled “rapid decline”. Finally, 8.1% of the population were labeled “poor function and moderate decline.”

**Table 3:** Latent classes mixed model fit parameter estimates for 1–4 classes using a linear function.

***N* classes**	***N* of parameter**	**AIC^b^**	**BIC^c^**	**Class parameter**	**1**	**2**	**3**	**4**
1	2	34,323.19	34,334.85	*N*	2,503			
				%	100			
				*APPA^*d*^*	1			
	3	34,323.85	34,338.41	*N*	2,503			
				%	100			
				*APPA^*d*^*	1			
2	2	32,081.46	32,098.94	*N*	959	1,544		
				%	38.31	61.68		
				*APPA^*d*^*	0.9442	0.9606		
	3	32,083.83	32,107.13	*N*	959	1,544		
				%	38.31	61.68		
				*APPA^*d*^*	0.9438	0.9610		
3	4	31,561.02	31,590.14	*N*	343	964	1,196	
				%	13.72	38.52	47.76	
				*APPA^*d*^*	0.9110	0.8935	0.9407	
	4	31,560.98	31,593.02	*N*	343	964	1,196	
				%	13.71	38.51	47.78	
				*APPA^*d*^*	0.9108	0.8934	0.9409	
4	7^a^	31,421.81	31,465.50	*N*	205	841	601	856
				%	8.18	33.61	24.01	34.20
				*APPA^*d*^*	0.8981	0.8114	0.8500	0.8821
4	8^a^	31,423.25	31,469.85	*N*	205	841	601	856
				%	8.15	24.00	33.62	34.23
				*APPA^*d*^*	0.8987	0.8504	0.8114	0.8822

a Preferred model.

b Akaike information criterion.

c Bayesian information criterion.

d Average posterior probability of assignment.

### Predictors of the physical function trajectory membership

**Figure 3:**
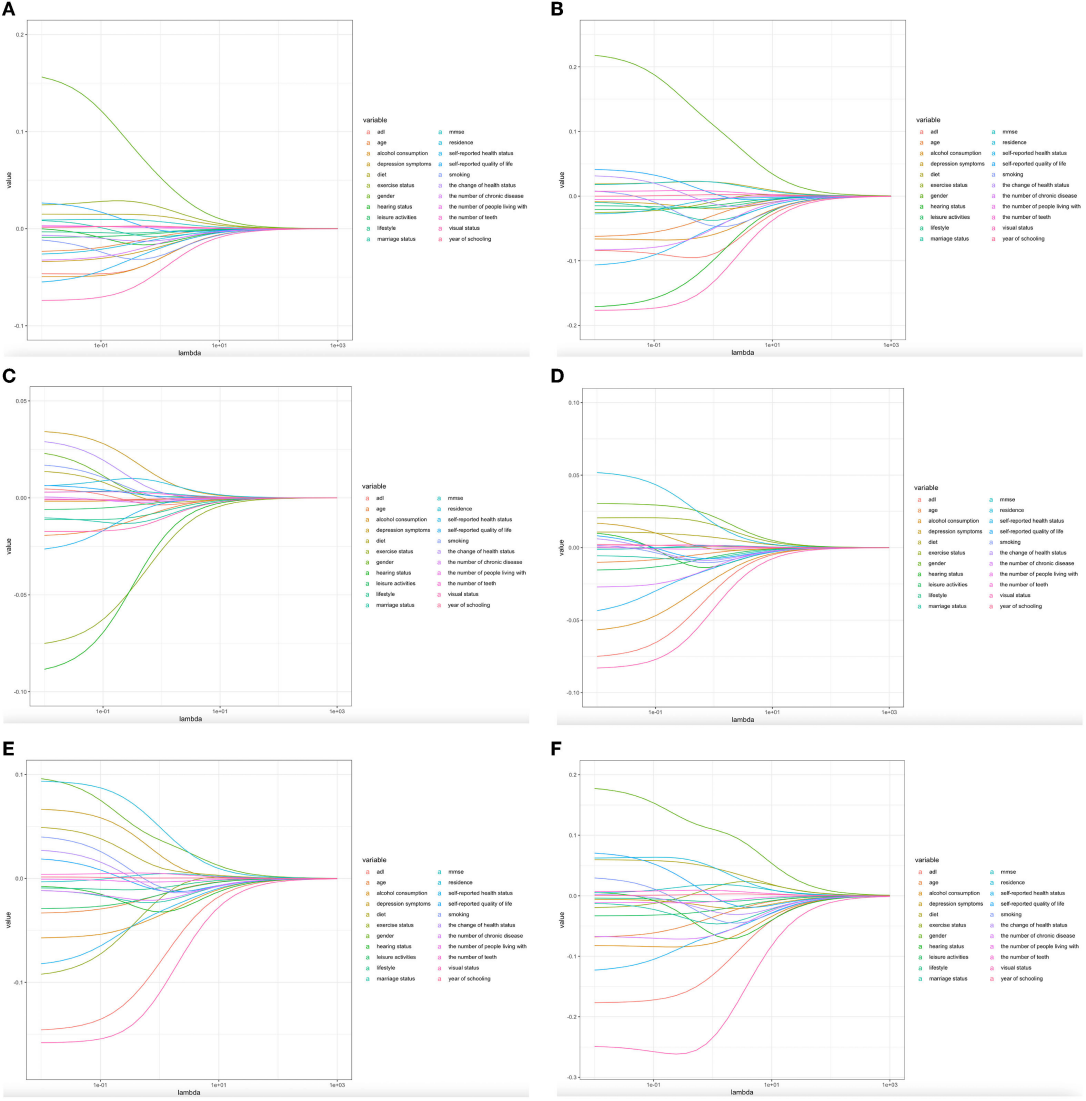
Predictors' selection using Lasso regression. **(A–F)** Lasso coefficient profiles of all the clinical features.

**Figure 4:**
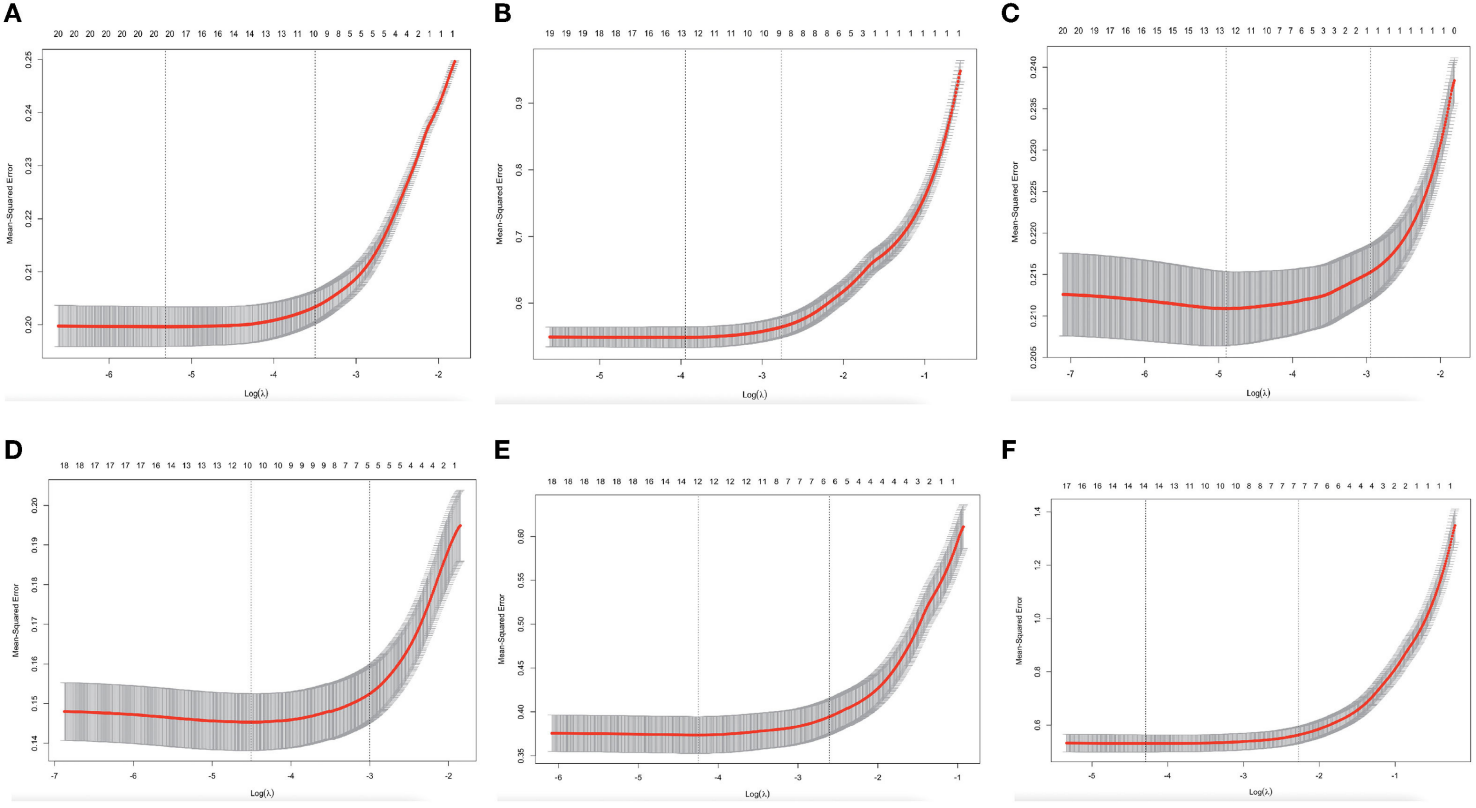
Predictors' selection using Lasso regression. **(A–F)** Identification of the optimal penalization coefficient λ in the Lasso model with 10-fold cross-validation and the minimum criterion.

We used the LASSO algorithm by the “Predict” function to explore the factors influencing the degree of decline in the different groups. To achieve more valuable clinical results, we have created six models. Model 1 (stable vs. slow decline), model 2 (stable vs. rapid decline), model 3 (slow decline vs. rapid decline), model 4 (rapid decline vs. poor function with moderate decline), model 5 (slow decline vs. poor function with moderate decline), and model 6 (stable vs. poor function with moderate decline). We determined that in model 1, all 19 variables remained in the model (i.e., not zero) when λ = 0.004915. In Figure 3, we show that 19 variables (age, sex, residence status, etc.) remained in the model the longest as physical function increases, with the remaining variables approaching zero more quickly. When the value was increased to 0.0322, only 11 variables, which may have an enormous effect on IADL scores, remained in the model. A comparison of our findings to those of others is shown in Figures 3, 4.

**Figure 5:**
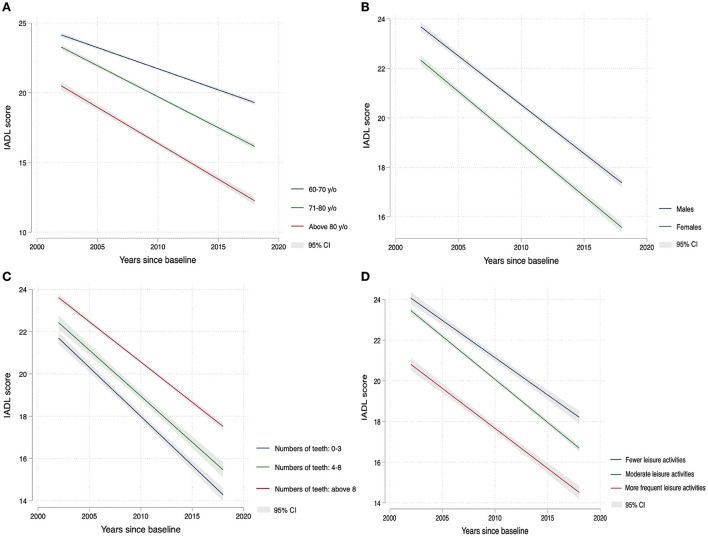
Linear variation of IADL scores with years of follow-up in different subgroups since the baseline: **(A)** age (y/o years), **(B)** gender, **(C)** the number of teeth, and **(D)** leisure activites.

Next, the above-incorporated variables were regressed using logistic regression. In the multivariate-adjusted multinomial logistic regression analyses, age, sex, self-reported health status, visual status, number of diseases, cognitive function, and leisure activity had statistically significant ratios (ORs) in both the slow and rapid decline groups relative to the stable group. Furthermore, compared to the stable group, depressed status (OR = 0.97, *p* < 0.001) and alcohol consumption (OR = 0.95, *p* < 0.01) contributed to the slow decline in physical function, while the number of teeth (OR = 1.01, *p* < 0.001) and hearing status (OR = 0.98, *p* < 0.05) may have increased the rate of decline. A comparison of the slow decline group with the rapid decline group revealed that age (OR = 0.98, *p* < 0.001), number of teeth (OR = 1.00, *p* < 0.05), depressive state (OR = 1.04, *p* < 0.05), exercise state (OR = 0.99, *p* < 0.001), and hearing state (OR = 0.91, *p* < 0.05) may accelerate the rate of decline when the slow decline group was used as a benchmark. More details are shown in Table 4. The two-way linear prediction plots of the changes in the IADL score with the follow-up years based on each determinant also demonstrated the same trend (Figure 5).

**Table 4:** Associations between different development trends of IADL.

**Baseline variable**	**Model 1**		**Model 2**		**Model 3**		**Model 4**		**Model 5**		**Model 6**	
	**OR**	**95% CI**	**OR**	**95% CI**	**OR**	**95% CI**	**OR**	**95% CI**	**OR**	**95% CI**	**OR**	**95% CI**
Age	0.98^***^	(0.97–0.98)	0.93^***^	(0.93–0.94)	0.98^***^	(0.97–0.98)	0.99^***^	(0.99–0.99)	0.97^***^	(0.96–0.97)	0.93^***^	(0.92–0.94)
Gender	1.18^***^	(1.10–1.23)	1.25^***^	(1.14–1.37)	1.03	(0.96–1.08)	1.04	(0.98–1.10)	1.09^*^	(1.01–1.19)	1.20^***^	(1.08–1.33)
Residence	0.97	(0.94–1.00)	/	/	/	/	1.05^**^	(1.01–1.09)	1.09^***^	(1.04–1.16)	1.06^*^	(1.00–1.13)
Number of nature teeth	1.00^*^	(0.99–1.00)	1.01^***^	(1.00–1.01)	1.00^*^	(1.00–1.01)	/	/	/	/	/	/
Self-reported quality of life	1.03	(0.99–1.05)			/	/	/	/	/	/	1.09^**^	(1.02–1.15)
Self-reported health status	0.94^***^	(0.91–0.97)	0.93^**^	(0.89–0.97)	0.97	(0.95–1.00)	0.97^**^	(0.95–0.99)	0.95^**^	(0.92–0.98)	0.88^***^	(0.83–0.94)
Depression symptoms	0.97^*^	(0.93–0.98)	/	/	1.04^*^	(1.00–1.07)	/	/	1.06^*^	(1.01–1.12)	/	/
The number of people living with	/	/	/	/	1.32	(1.00–1.07)	/	/	/	/	/	/
The marriage status	1.01	(0.99–1.03)	/	/	0.99	(0.97–1.01)	/	/	0.99	(0.96–1.02)	0.98	(0.95–1.02)
Change in self-perceived health status	1.00	(0.99–1.01)	/	/	/	/	/	/	/	/	0.99	(0.97–1.01)
Years of schooling	1.00	(0.99–1.00)	/	/	/	/	1.00	(1.00–1.01)	/	/	/	/
visual status	0.93^*^	(0.87–0.98)	0.83^***^	(0.75–0.93)	0.98	(0.93–1.04)	0.92^***^	(0.88–0.96)	0.85^***^	(0.80–0.91)	0.78^***^	(0.71–0.86)
Smoking	0.99	(0.93–1.04)	/	/	/	/	/	/	/	/	/	/
Alcohol consumption	0.95^*^	(0.90–0.99)	0.92	(0.84–1.00)	/	/	0.95	(0.88–1.02)	0.94	(0.85–1.03)	0.93	(0.84–1.03)
Exercise	1.02	(0.97–1.07)	/	/	0.91^***^	(0.87–0.96)	/	/	0.91^*^	(0.82–0.98)	/	/
Lifestyle	/	/	/	/	/	/	/	/	/	/	/	/
MMSE	1.00^*^	(1.00–1.01)	1.02^*^	(1.00–1.03)	1.00	(1.00–1.01)	/	/	/	/	1.01	(0.99–1.02)
ADL	0.95	(0.89–1.01)	0.92	(0.81–1.04)			0.93^***^	(0.90–0.95)	0.86^***^	(0.83–0.90)	0.84^***^	(0.80–0.88)
Leisure activities	0.99^***^	(0.98–0.99)	0.98^***^	(0.97–0.99)	0.99^*^	(0.99–1.00)	0.98^***^	(0.98–0.99)	0.97^***^	(0.96–0.98)	0.97^***^	(0.96–0.98)
Diet	1.01	(0.96–1.05)	/	/	/	/	/	/	/	/	1.06	(0.96–1.17)
Hearing status	/	/	0.85^*^	(0.72–1.00)	0.91^*^	(0.83–0.99)	/	/	/	/	/	/
The number of chronic disease	0.97^**^	(0.94–0.99)	0.92^***^	(1.88–0.96)	/	/	0.97^*^	(0.95–1.00)	0.98	(0.95–1.02)	0.93^**^	(0.89–0.98)

* p < 0.05.

** p < 0.01.

*** p < 0.001.

Model 1 (stable vs. slow decline), model 2 (stable vs. rapid decline), model 3 (slow decline vs. rapid decline), model 4 (rapid decline vs. poor function with moderate decline), model 5 (slow decline vs. poor function with moderate decline), model 6 (stable vs. poor function with moderate decline).

### Sensitivity analysis

A receiver operating characteristic (ROC) curve analysis was performed to assess the sensitivity and specificity of this risk prediction model, and we calculated the AUC to validate the precision of the established risk prediction model (Appendix A1). We removed the missing values from the dataset, and the remaining 769 values were subjected to a repeat analysis and yielded similar results (Appendix A2).

## Discussion

To the best of our knowledge, this is the first study to analyse physical function trajectories using data from a 16-year longitudinal study and explore their predictors using LASSO regression. This modern, robust statistical technique minimizes multicollinearity between variables. Our findings indicated four different trajectories of physical function in older people, namely, stable state (35.4%), slow decline (33.0%), rapid decline (23.5%), and poor function and moderate decline (8.1%). We found several characteristics that can be used to predict a decline in physical capacity. Regarding sociodemographic characteristics, age, sex, leisure activities, self-reported health status, worse vision status, more chronic diseases, worse cognitive function, and a decreased frequency of leisure activity influence the trajectory of physical function. Worse mental health and increased alcohol consumption are predictors of slow decline, whereas fewer teeth and worse hearing states are predictors of rapid decline. In terms of the rate of decline, using the slow decline group as a benchmark, fewer teeth, stronger depressive symptoms, lack of exercise, and reduced hearing may increase the rate of decline.

The pattern of identified trajectories varies across studies. These variations may be due to differences in the study populations, sample sizes, methods used to identify trajectories, and assessment schedules. A study of ADL trajectories in younger adults in the US revealed five cohorts, but most participants had good functional health, with 8% of subjects losing physical function after 10 years (33). In addition, the results of a 22-year cohort study from 1988 in two administrative regions of southwestern France showed that five different characteristics of physical decline were identified in the past two decades of life: persistently high (12%), moderate (26%), persistently low (40%), accelerated high dependence (15%), and no dependence (8%) (34). In contrast, one notable finding of our study was that more than half of the sample (69.1%) showed a sustained or slow decline in physical function throughout the 16 years, with only a minority showing a dramatic decline, slightly less than that in Arlette Edjolo's study. The possible reason for this is that the CLHLS contains national longitudinal data covering 32 geographically wide and representative regions.

Our results of the association of well-established physical function risk factors, including age, sex, education level, and multiple chronic diseases, are consistent with those of other studies (7, 34–36). Age and sex remained in the model and were strongly related to physical functioning decline. Cross-sectional data from a longitudinal study of Irish aging (TILDA) showed that marital status was a predictor of IADL, but this result was not found in our study (37). More people with poor marital status were excluded from our baseline population. With an increasing aging population, it would be expected that the prevalence of IADL disabilities will increase and maintaining the physical function of older people has become an urgent concern. In 2017, the WHO published guidelines for integrating caregiving for older adults and suggested that intrinsic capacity is key to preventing and slowing disability and promoting healthy aging (38, 39). IADL can be used to evaluate the overall intrinsic capacity, but researchers should be aware of and address the modifiable factors associated with IADL.

In contrast, our results also demonstrated that the number of teeth is another risk factor for physical function limitations. Oral health, especially tooth loss, is considered an early indicator of physical function limitations and is often overlooked in research. Our study showed that the number of teeth was associated with a trajectory in both the contrast stable and slow decline groups (OR = 1.00) and in the stable and rapidly declining groups (OR = 1.01). Furthermore, fewer teeth increased the rate of decline in physical function (OR = 1.00). A cross-sectional study from the United States that surveyed 114,602 Americans aged 65 years and older found that the number of teeth lost was significantly associated with PFL. Similarly, those who have lost six or more teeth but not all may have PFL (40). The same results were also found in other studies (41, 42). Nutritional status may be related to the mechanism of tooth loss associated with physical condition (43). It is well known that adequate protein intake helps to limit and treat age-related declines in muscle mass, strength, and functional capacity and prevents the onset of frailty (44).

Another interesting result is that we found that changes in perceptual function (including vision and hearing status) were positively correlated with changes in physical function. The possible reason for this finding is that hearing loss leads to a possible reduction in an individual's ability to communicate, reducing their social participation and thus IADL (45, 46). A geriatric assessment includes an evaluation of an individual's hearing and vision, which are key components of older adult health (47). In the 2015 Global Burden of Disease Study (GBDS), visual impairment and hearing loss were the second and third leading causes of various impairments, respectively (48). Moreover, dual sensory impairment is associated with a higher risk of all-cause mortality than a single impairment (49, 50). Hearing loss may lead to decreased physical function through several possible pathways. There are several hypotheses, first, that movement may be dependent on sound input from the external environment and that hearing loss may reduce the ability to perform complex movements effectively (51). Second, factors such as social participation and mental health are mediating variables between hearing loss and physical function (52–55). Also, more indirect confounding pathways may exist (56).

In addition, our results showed that older adults who were less involved in leisure activities such as playing cards, watching TV, and working in the garden were more likely to experience and rapidly develop physical functional limitations, with significant differences in all three models. This suggests that leisure activities have a protective effect on physical function. Additionally, in the first and third models, we found that depression positively influenced IADL, similar to the self-rated health status in the first and second models. According to previous reports, depression affects physical function limitations, while leisure activities improve social participation, enhance cognitive function, and increase mental health status (29, 57). We conjecture that the mechanism is that depressive symptoms and the self-rated health status are mediating factors of leisure activities and IADL levels.

It is important to note that many previous studies have emphasized that lifestyle strongly affects physical function (22, 58–60). However, in our study, alcohol consumption showed a positive correlation, and the results were insignificant in Model 3. This may be due to our inaccurate definition of diet, and many studies are now constantly seeking the optimal components of a healthy diet. In addition, in terms of exercise, its frequency and intensity may affect the outcomes (61), and future prospective studies could further explore exercise.

The strengths of our study include, first, the large sample size provided through the CLHLS. The CLHLS provides sufficient power to identify trajectories and find differences between them. The results are generalizable due to the national representativeness of the CLHLS data. Second, in this study, we used a GBTM, which can identify clusters of individuals who follow similar developmental trajectories on a given outcome by fitting a semiparametric mixture model to longitudinal data to maximize the quality of the data. Third, we used variables that were filtered by LASSO regression to be incorporated into a logistic regression model to improve the test efficacy of logistic regression and increase the significance of the variables.

The results of this study should be interpreted with some caution. First, the variables we used were derived from self-reported surveys, which may lead to bias. However, self-reported data are commonly used in physical status studies of older adults and can more accurately reflect the statuses of individuals interacting with the real world. Second, we used multiple imputation (MI) approach to handling attrition and missing data, the sensitivity analysis was also done, however, bias due to the other sources of potential threats could still not be completely avoided. Third, causality could not be determined due to the current cohort design. Given that a decline in physical function is a long-term process, although the 16-year cohort has been considered current, the length of the study is relatively long for a physical function analysis, and this may have resulted in an underestimation of the number of trajectories.

## Conclusions and implications

Overall, this study shows four trajectories of physical function that were identified in a national, 16-year follow-up sample of community-dwelling older adults. Declining physical function results from a multifactorial process that includes sociodemographic characteristics, psychosocial factors, and lifestyle factors. Our study used a machine learning approach to identify variables that are more significant to the trajectory of physical function, and the accuracy of the six risk models was verified using ROC curves. Currently, many scholars focus on early prevention and timely intervention for age-related problems. This research helps maintain or slow the rate of decline in body function and improves healthy aging.

## Data availability statement

Publicly available datasets were analyzed in this study. This data can be found at: https://charls.charlsdata.com/pages/data/111/zh-cn.html.

## Ethics statement

The studies involving human participants were reviewed and approved by Peking University (IRB00001052-13074). The patients/participants provided their written informed consent to participate in this study.

## Author contributions

Systematic concept and designed: YZ, YD, HF, and LX. Analysis the data: YZ, JN, and XL. Drifting the manuscript: YZ and YD. Revised the manuscript, read, and approved the submission of this manuscript: all authors.

## Funding

This work was supported by the National Key R&D Program of China (Grant Number 2020YFC2008602) and the Natural Science Foundation of China (Grant Number 72174212), and the National Key R&D Program of China (Grant Number 2020YFC2008503).

## Conflict of interest

The authors declare that the research was conducted in the absence of any commercial or financial relationships that could be construed as a potential conflict of interest.

## Publisher's note

All claims expressed in this article are solely those of the authors and do not necessarily represent those of their affiliated organizations, or those of the publisher, the editors and the reviewers. Any product that may be evaluated in this article, or claim that may be made by its manufacturer, is not guaranteed or endorsed by the publisher.

## Figures and Tables

**Figure A1:**
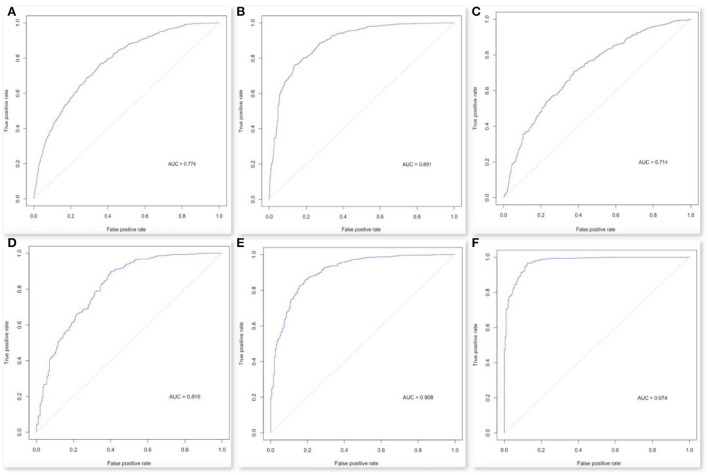
ROC in Lasso regression. The ROC scores range in six models. **(A)** model 1, **(B)** model 2, **(C)** model 3, **(D)** model 4, **(E)** model 5, and **(F)** model 6.

**Figure A2:**
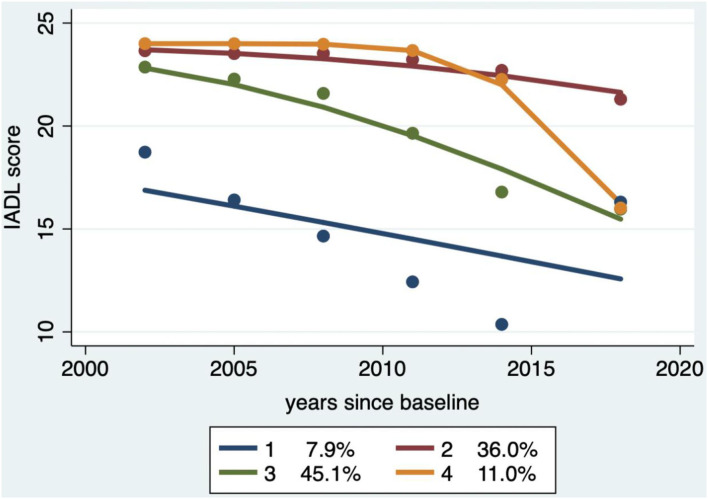
Results of physical function trajectories in 769 older adults.
